# The Interactive Construction of Biological Individuality Through Biotic Entrenchment

**DOI:** 10.3389/fpsyg.2019.02578

**Published:** 2019-12-02

**Authors:** Isaac Hernández, Davide Vecchi

**Affiliations:** ^1^Laboratoire de Recherche ERRAPHIS, Département de Philosophie, Université Toulouse Jean Jaurès, Toulouse, France; ^2^Centro de Filosofia das Ciências, Departamento de História e Filosofia das Ciências, Faculdade de Ciências, Universidade de Lisboa, Lisbon, Portugal

**Keywords:** autopoieisis, biological autonomy, philosophy of biology, biological individuality, emergent evolution, biological identity, symbiosis, individuation

## Abstract

In this article, we propose to critically evaluate whether a closure of constraints interpretation can make sense of biotic entrenchment, the process of assimilation and functional integration of environmental elements of biotic origin in development and, eventually, evolution. In order to achieve the aims of our analysis, we shall focus on multi-species partnerships, biological systems characterised by ontogenetic dependencies of various strengths between the partners. Our main research question is to tackle the foundational problem posed by the dynamics of biotic entrenchment characterising multi-species partnerships for the closure of constraints interpretation, namely, to understand for which biological system (i.e., the partners taken individually or the partnership as the encompassing system) closure of constraints is realised. Through the analysis of significant illustrative examples, we shall progressively refine the closure thesis and articulate an answer to our main research question. We shall also propose that biotic entrenchment provides a chief example of the phenomenon of interactive and horizontal construction of biological individuality and inter-identity.

## Biological Individuality Between Closure and Entrenchment

The characterisation of the criteria for the individuation of developing and evolving living entities is one of the main issues in the philosophy of biology and theoretical biology. From a Darwinian perspective, based on the notion of unit of selection, organisms represent just one individual amongst many possible types. This notion should be contrasted to that of physiological individual focused on functional integration^1^. The autopoietic approach is an important instance of the latter. Autopoiesis, as its name suggests (a term with Greek etymology from *auto* = self and *poiesis* = production), is a theory characterising organismal life in terms of the self-maintenance of organisation through some form of *self-production*. Autopoiesis characterises the individual as a unit of organisation to be understood in terms of the processes of self-distinction through which the constituent parts of the biological system generate an autonomous domain of relations: an operational closure (Maturana and Varela, 1991). The limits of the individual are thus the limits of this closure, which becomes the fundamental criterion for tracing the boundary between individual and environment. In our view, the chief challenge faced by this approach is to provide a characterisation of the concept of closure that accounts for interactive biological dynamics like entrenchment (West-Eberhard, 2003). West-Eberhard emphasises a neglected but at the same time fundamental process in biology: the role of the environment in the regulation of development and in the production of the phenotype. In the first sense, environmental factors can serve as signals or cues at switch points (i.e., the bifurcations paving the developmental pathway, see Vecchi et al., 2019 for an analysis of the switch-model of development). In the second sense, environmental “materials” can serve as building blocks in phenogenesis (i.e., phenotype construction or formation). In both cases, initially persistent, unavoidable, recurrent environmentally-supplied elements (i.e., signals and materials) can become essential for normal development, resulting in entrenchment, that is, in the establishment of ontogenetic and evolutionary dependencies on environmental elements on the part of biological organisms (West-Eberhard, 2003, pp. 500–503). Given our focus on ontogeny, by entrenchment (see section “The Entrenchment of Environmental Elements in Ontogeny”) we will refer to the process of assimilation and functional integration of environmental elements, particularly the establishment of ontogenetic dependencies on environmental elements on the part of biological organisms^2^. Entrenchment is thus a process of integration of elements heterogeneous to the biological system’s internal organisation through ontogeny and, eventually, evolution. Significantly, these environmental elements can be biotic in origin rather than the result of chemical processes (e.g., the chemical elements or other precursors required for protein synthesis). *Biotic entrenchment* occurs when the environmental elements are produced by other organisms or, at the extreme, when such elements are other organisms themselves (e.g., endosymbionts). In this sense, biotic entrenchment provides a chief example of the phenomenon of interactive construction of individuality and inter-identity. As the editors of this thematic issue suggest, complex biological systems display not only vertical complexity (i.e., the hierarchical organisation of parts making up a whole), but also “horizontal” organisation, where this latter organisational dimension influences, on a developmental and, ultimately, evolutionary time scale, their biological identity. In this sense, biotic entrenchment is a process of horizontal generation of organisation. The vast and growing literature on multi-species aggregates (Queller and Strassmann, 2016) such as biofilms (Ereshefsky and Pedroso, 2015), holobionts (Skillings, 2016), and hybrids (Chiu and Eberl, 2016) focuses on the putative individuality of a variety of multi-species *partnerships*. These partnerships are all products of what we call biotic entrenchment. This literature shows the increasing attention biology and philosophy of biology are paying towards the emergence of inter-identity, the dynamics of horizontal organisation and the interactive construction of individuality through entrenchment. The nature of multi-species partnerships is extremely varied. They range from environmentally induced mutualistic associations (Hom and Murray, 2014) to complex host-endosymbiont relationships (Hehemann et al., 2010), from virally-triggered biofilm formation (Fernández et al., 2017) to outsourcing of developmental signals (Gilbert et al., 2010; Selosse et al., 2014)^3^, from microbiota-mediated enzyme production (Lu and Walker, 2001) to construction of extracellular organs for the exchange of nutrients and enzymes (Corradi and Brachmann, 2017), etc. In all these cases, ontogenetic dependencies are established between the associated organisms that might translate into stable evolutionary ones. The interplay between developing ontogenetic dependencies and evolving stable evolutionary ones is the crux of the problem of biological individuality from both a physiological and evolutionary perspective^4^. In this sense, the challenge faced by the autopoietic approach is to reconcile the notion of closure with the ubiquity of the functional exchanges characterising the biotic entrenchment represented by multi-species partnerships.

### Closure and Entrenchment

The notion of closure is crucial within the autopoietic framework of analysis because it provides a putative clear-cut criterion for distinguishing the identity of the system with respect to other systems and the environment: the limits of the biological individual are the physical or functional borders (see section “Can Components Assimilated From the Environment Become Integrated in the Functional Organisation O and Perform a Function?” for an analysis of this distinction) of the biological organisation represented by organisational closure. Autopoietic systems are thermodynamically open but organisationally closed. On the one hand, autopoietic systems are open materially and energetically. The material and energetic openness to the environment ensures the circulation of the energy and matter necessary for the maintenance of the organisation. On the other hand, biological systems are organisationally closed. The closure thesis can be interpreted in many ways (some of them reviewed in section “A Characterisation of Closure of Constraints in the Light of Entrenchment”) that, at a first approximation, share the insights that the system somehow determines itself and that the biological identity of the system depends on some aspect of its organisation. Thus, whilst an autopoietic system depends on its physico-chemical milieu (because it is thermodynamically open), it is also identified as distinct from its environment. In this sense, there is a crucial difference between an autonomous internal domain of relations, self-determined by the organism, and the environment with which it exchanges energy and matter. As a result, one of the possible interpretations of the autopoietic approach is that biological individuality can be conceptualised without taking into account the organisational and functional roles played by the environment in the construction and maintenance of biological organisation. Within this context, the examples of entrenchment that we discuss in section “The Entrenchment of Environmental Elements in Ontogeny” seem to be amenable to a different kind of analysis.

Our aim in this article is to critically evaluate one of the crucial insights stemming from the autopoietic tradition, namely the idea that closure, despite the variability of its concrete realisations, is a fundamental “invariant” of biological organisation. The rationale of this insight is that, without some form of closure, a biological system would be just a cluster of unconnected processes and reactions. Our analysis will focus on the *closure of constraints* interpretation (Montévil and Mossio, 2015; Moreno and Mossio, 2015). The basic idea of this interpretation is to distinguish two “regimes of causation”, one involving processes and another involving constraints. Constraints can be characterised as higher-level structures reducing the degree of freedom of lower-level components within an orchestration of components or organisation. Constraints thus account for some inter-level causal relations and make sense of the notion of control by higher-level structures on the lower-level components in the same organisation. In this sense, they are a possible solution to the puzzle of explaining how wholes exert control on their constituent parts and, particularly, of explaining how biological systems like organisms are dual-control systems with an autonomous harnessing principle (see Umerez and Mossio, 2013). In this latter sense, constraints make sense of the notion of control by intrinsic rather than external structures within an organisation (what the literature calls *constitutive constraints*, Montévil and Mossio, 2015). They thus ground the idea of autonomous self-maintenance. Pattee (see Winning and Bechtel, 2019) used the notion of constraint in order to refer to structures that control the behaviour of lower-level components; this control is exerted not because of the dynamical interaction of structures with lower-level components, but rather because constraints channel the components’ behaviour along a limited set of routes. In this sense, constraints are material macro-structures selectively limiting the degrees of freedom of micro-components (Moreno and Mossio, 2015, pp. 12–13, note 20). Constraints are such only at a relevant temporal and spatial scale and relative to a specific thermodynamic flow, material input, or process^5^. Thus, constraints are, relative to a certain scale, conserved with respect to the components. It is because of this relative stability and conservation that they can exert their causal powers by channelling components’ behaviour. Furthermore, given that constraints are just characterised as macro-structures with respect to some lower-level components, they can be structures at many levels of biological organisation. For instance, enzymes constrain chemical reactions in the sense that they harness the behaviour of the material inputs or chemical components of a metabolic reaction as well as the rate of the reaction (Montévil and Mossio, 2015, p. 183). Metabolic pathways constrain chemical reactions: in the urea cycle, the cycle of chemical reactions is constrained in the sense that the behaviour of reactants proceeds along a specific cyclic and recursive route: in a simplified form, ornithine reacts with ammonia to produce citrulline, then citrulline reacts with aspartate to produce arginine, then arginine reacting with water decomposes to urea and ornithine, where the latter is used as a starting component of the same cyclic reaction. Metabolic pathways also constrain the behaviour of enzymes in the sense that a particular enzyme can only perform a specific function within the context of the pathway. For instance, in the Calvin-Benson cycle performed by phototrophs, the enzyme RuBisCo can only perform a specific functional role that is determined by the topological features of the macro-structure or metabolic pathway itself. The circulatory system constraints the movement or circulatory behaviour of nutrients, oxygen, carbon dioxide, hormones, blood cells, etc. All such micro-components can only follow specific paths determined by the macro-structure of the vascular system (itself constituted of an organisation of cells with specific geometrical and topological properties) instead of diffusing throughout the body of the organism (Montévil and Mossio, 2015, p. 189). Thus, whilst biological systems are materially and energetically open and undergo continuously a variety of ontogenetic structural and functional changes, they nonetheless display some form of stability in a specific organisational sense identifiable as a closure of constraints, i.e., a self-maintaining pattern of mutual dependence between their constitutive constraints. As we shall argue in sections “A Characterisation of Closure of Constraints in the Light of Entrenchment” and “A Characterisation of Organisational Invariance in the Light of Entrenchment,” the advocates of the closure of constraints interpretation have made a number of adjustments in order to make closure compatible with the biological reality of developmental change and the dynamics of functional variation. However, biotic entrenchment poses an additional kind of challenge. At the heart of the problem is the distinction between internal or constitutive constraints (those realised within the spatial boundary of the biological system’s organisation) and external ones (those realised between the biological system and the environment; for instance, through biotic entrenchment). When the organisms in a multi-species partnership develop irreversible ontogenetic mutual dependencies such that they rely on the partners for their own physiological maintenance (that is, when their self-maintenance is not reducible to the internal system of constraints realising closure but also depends on the existence of external constraints), it might be argued that the individual partners do not realise closure. It thus becomes difficult to discriminate for which biological system closure is realised: suppose that two organisms A and B start sharing a functional constraint through biotic entrenchment; what is the closed biological system? A, B, or the partnership C between A and B? When partners establish irreversible ontogenetic dependencies, how can each partner be individuated as that very same biological system independently of the interactions with others? As we shall see in section “Can Components Assimilated From the Environment Become Integrated in the Functional Organisation O and Perform a Function?”, a possible solution to this puzzle can be drawn from extended autopoietic accounts based on closure (Virgo et al., 2011). This is, in our opinion, the crux of the problem posed by entrenchment for autopoietic accounts based on closure. Indeed, Montévil and Mossio (2015, p. 188) acknowledge that failure to solve this theoretical problem implies a weakness for any account based on closure. Whilst Montévil and Mossio (2015) address this problem in an abstract way, in this article we propose to analyse the empirical cases of entrenchment illustrated in section “The Entrenchment of Environmental Elements in Ontogeny” at a fine-grained level of detail. These examples are tailored to critically assess the biological feasibility of the notion of closure of constraints, test whether indeed this notion is compatible with entrenchment and eventually open up a new perspective to understand biological individuality. We have chosen these examples for two reasons. The first is that, in order to answer the research question concerning the compatibility between entrenchment and closure, we need to consider examples that are amenable to analysis in terms of “developmental events^6^.” Even though biotic entrenchment is, ultimately, an evolutionary phenomenon, our analysis aims to reconstruct this evolutionary history in terms of the developmental events occurring to the relevant partners, without taking into consideration inheritance^7^. Put differently, our analysis emphasises the ontogenetic dependency between partners. A second reason to choose our examples has to do with their variety: entrenchment between unicellular, multicellular organisms, and other biological systems are illustrated^8^. It might thus be objected that the analysis we propose in terms of developmental events is not feasible, given that unicellular organisms do not develop, at least in the classical sense of development (Nuño de la Rosa, 2010, p. 292). Development can be characterised in many ways, some restrictive and some less so. We favour the latter avenue and characterise development, following West-Eberhard (2003, pp. 89 ff), as the series of phenotypic and qualitative changes a responsive biological system undergoes due to environmental and genomic inputs. If development is characterised in these general terms, every organism is capable of development if it undergoes phenotypic qualitative changes during its life history^9^. In this light, the examples of entrenchment we illustrate in the next section can be analysed in terms of significant developmental events such as genome reduction (section “Dependence Through Division of Metabolic Labour”) or lateral gene transfer (section “Mutual Dependence Through Genomic, Metabolic, and Cellular Integration”) or the incorporation of an externally produced enzyme in a metabolic pathway (section “Mutual Dependence Through Genomic, Metabolic, and Cellular Integration”).

## The Entrenchment of Environmental Elements in Ontogeny

As we related in section “Biological Individuality Between Closure and Entrenchment,” West-Eberhard emphasises a neglected but at the same time fundamental process in biology: the role of the environment in the regulation of development and in the production of the phenotype. In both cases, initially persistent, unavoidable, recurrent environmentally-supplied elements (i.e., signals and materials) can become essential for normal development, resulting in entrenchment, that is, in the establishment of opportunistic ontogenetic and evolutionary dependencies on environmental elements on the part of biological organisms (West-Eberhard, 2003, pp. 500–503). For instance, in a very fundamental sense, gene expression cannot be performed without environmental elements. For example, mammals are unable to synthesise all amino acids necessary for protein biosynthesis; what mammals do is to get them from nutrition, by eating the organisms that actually synthesise them (e.g., bacteria and plants) and then extracting already functional molecular components; without such environmentally-supplied elements, mammals would not be able to effectively perform gene expression, for instance the biosynthesis of haemoglobin. The same point can be made about many aspects of metabolism: humans are also unable to synthesise various vitamins so that they must assimilate them through nutrition; birds’ digestion depends on the ingestion of stones that function as gastroliths; oxygen and sunlight are other recurrent environmentally-supplied elements of animal and plant development, respectively, etc. As West-Eberhard (2003, p. 500) puts it, all these elements are entrenched, with the consequence that: “None of these essential components of the phenotype emanate from the genome. Indeed, nothing emanates from the genome without environmental materials…”.

West-Eberhard’s analysis is instrumental to support her hypothesis that phenotypic novelty is often environmentally initiated rather than being caused by internal genomic change or self-generated. For our analysis, the implications of entrenchment are various. First of all, to think about the environment as merely posing challenges and disturbances to which organisms must respond and resist in order to survive and preserve their identity is clearly at odds with the *constructive* role the environment plays in ontogeny. Focusing on metabolism, entrenchment makes it clear that the self-production claim often associated with the autopoietic tradition should be appropriately qualified (see section “Can Set S Be Extended by Assimilating Components From the Environment?”). The reason is that there is arguably no living organism that self-produces *all* the components it requires to maintain its identity in ontogeny. Does a unicellular organism self-produce, for instance, all its DNA sequences and all the molecular components for protein biosynthesis? The ubiquity of lateral gene transfer and the assimilation of a variety of chemical precursors make the strict self-production scenario at best unrealistic. When other kinds of organisms are considered, we know that many of them have lost self-production capacities in multifarious senses, for instance in terms of the inability of synthesising various amino acids or vitamins. This points to a second crucial aspect of entrenchment. Organisms rely on the entrenchment of environmental elements that are not only abiotic in origin (e.g., produced by chemical processes and at the basis of the assimilation, for instance, of the precursors required for amino acid and protein syntheses, a phenomenon that we could call *chemical entrenchment*); in fact, often environmental elements are biotic in origin: many kinds of organisms rely on the production capacities of other organisms. Indeed, the loss of organismal capacities of self-producing various molecular components is explained by this *reliance on other organisms*, by a primitive form of cooperation, horizontal organisation or inter-identity. Crucially, the assimilated elements might also be deployed in order to perform new functions. Indeed, whole organisms (e.g., symbionts) may act as entrenched environmental elements in development and metabolism: symbiont-partners may be assimilated by host-partners, as all eukaryotic cells and holobionts show (see section “Mutual Dependence Through Genomic, Metabolic, and Cellular Integration”). The entrenchment of biotic environmental elements captures a widespread biological phenomenon: *the outsourcing of components’ production* to other organisms. As West-Eberhard’s makes clear, this is not surprising given that an organism’s environment includes other organisms. As anticipated in section “Biological Individuality Between Closure and Entrenchment,” we call this phenomenon *biotic entrenchment*. In brief, the logic of biotic entrenchment is that the environment has a constructive role in ontogeny in two senses: on the one hand, components’ production can be outsourced to other organisms and, on the other, outsourced components might be deployed to generate new functions. Thus, as we shall see in section “A Characterisation of Closure of Constraints in the Light of Entrenchment,” the problem faced by autopoietic approaches is to reconcile the notion of closure with biotic entrenchment.

Before proceeding with the analysis of the examples of biotic entrenchment, a clarification is required. It is useful to distinguish between the process of entrenchment on the one hand, and the outcome of the process. This terminological distinction mirrors that concerning, for instance, the term “adaptation,” which might be used to refer, confusingly, to the process of adaptive evolution and to its phenotypic outcome. The process of entrenchment can be decomposed in the process of assimilation of environmental elements and that of their functional integration. These two processes have an ontogenetic and evolutionary dimension. Entrenchment as an outcome is the emergence of a partnership and, ultimately new biological individual, as a result of the process of entrenchment. This distinction will be useful for the analysis in sections “Can Components Assimilated From the Environment Become Integrated in the Functional Organisation O and Perform a Function?” and “A Characterisation of Organisational Invariance in the Light of Entrenchment.”

### Dependence Through Division of Metabolic Labour

Let us examine our first example. Some extremely abundant marine species of bacteria (e.g., *Prochlorococcus marinus* and *Candidatus Pelagibacter ubique*, henceforth Ps) lack genes to survive oxidative stress, specifically genes (*katG*) coding for enzymes (catalase-peroxidase) that are necessary to resist HOOH (i.e., external hydrogen peroxide, H_2_O_2_). The metabolic importance of such genes is hard to underestimate, given that HOOH is capable of killing axenic *Prochlorococcus* cultures in a few hours (Morris et al., 2012). But these species underwent a process of genome reduction. Given that HOOH removal and water detoxification is so crucial for the survival of these dominant bacteria, why did genome reduction happen? The answer is that these groups of organisms (i.e., Ps) depend on other organisms for the reduction of HOOH (henceforth Hs):

“…the loss of HOOH resistance can be described as a community-dependent adaptive event. It is adaptive because resources may be shunted from HOOH defense to growth, but only because other members of the community condition the environment such that a robust oxidative-stress response becomes dispensable to the beneficiaries.” (Morris et al., 2012, p. 2)

The evolution of this functional dependency in marine microbial communities is based on the principle that metabolically costly functions might be abandoned if other organisms produce “public goods” and benefits for the ecosystem. This pattern of division of metabolic labour is very common. For instance, nitrogen fixation – a metabolic function very demanding energetically – is solely performed “… by a relatively small subset of organisms; for example, in the oceans, nitrogen-fixing species (diazotrophs) constitute less than 1% of the total cyanobacterial population.” (Morris et al., 2012, p. 4). This example of biotic entrenchment epitomises the principle of outsourcing of components’ production. In other words, it is not necessary for Ps to produce all the essential metabolic resources; rather, what is necessary is to possess them at the right time, independently of how they are produced and acquired. The stability of the partnership depends on the constant renewal, at each generation, of a *specific functional coupling* between Ps and Hs, i.e., the organisms performing HOOH removal. Thus, this dependence is reinforced through ecological coevolution. Also note that, even though the dependence is evolutionary reversible (if Ps regain the ability to detoxify marine water by mutation or by lateral DNA transfer), it is *ontogenetically irreversible* in the sense that Ps cannot survive without Hs.

Two developmental events characterise the establishment of the ontogenetic dependency between Ps on Hs. First, genome reduction is a developmental event in the life of Ps that causes the loss of their ability to biosynthesise catalase-peroxidase enzymes. It is a developmental event because gene loss happens to single organisms. It is only after such events that a population effect ensues (i.e., that the majority of members of the population do not possess the *katG* genes). Secondly, Ps must be able to assimilate deoxygenated water and make use of this resource in their metabolism. This is not, obviously, a major problem given that all organisms can effortlessly use deoxygenated water as a metabolite, as a solvent, as a structural component of the aqueous environment of protein folding etc., that is, to perform metabolic roles in a vast range of chemical reactions. The functional integration is therefore, by assumption, straightforward because it does not require a major reorganisation of the physiology of Ps. The same can be said about the assimilation of DNA molecules through lateral DNA transfer, which many organisms (e.g., bacteria) are able to easily functionally integrate because such integration does not require, for instance, the reorganisation of the molecular apparatus of transcription. What is rather important in this sense is the “simplification” of metabolism, namely, the fact that Ps will not need to make use of the metabolic pathway where hydrogen peroxide is a reactant. This simplification is, in the language of closure of constraints, a loss or a suppression of a constraint.

### Mutual Dependence Through Genomic, Metabolic, and Cellular Integration

The green sea slug *Elysia chlorotica* assimilates chloroplasts by feeding on the marine algae *Vaucheria*. It is thus a “photosynthetic” animal. The partnership between *Elysia chlorotica* and two *Vaucheria* species is both specific (*Elysia chlorotica* only associates with these two species of *Vaucheria*, i.e., *V. litorea* and *V. compacta*) and obligate (the sea slug would not fully develop without *Vaucheria*). However, no reproductive co-transmission ensues (i.e., no sea-slug-algae coordinated inheritance system has evolved allowing the vertical transmission of the plastids to the sea slugs of the offspring generation). In fact, the slug needs to assimilate the plastids at each generation by eating the marine algae *Vaucheria*, making this a developmentally and metabolically significant dependency that is particularly apt for our analysis. The *Vaucheria*’s chloroplasts are absorbed by phagocytosis and then sequestered in the specialised digestive tubular cells in the slug’s gut, where they are kept functional. Indeed, this slug is able to perform photosynthesis *via* the chloroplasts that it “steals” from *Vaucheria litorea* (a phenomenon called kleptoplasty). As a consequence, the sea slug can survive several months – satisfying its nutrients needs – solely by photosynthesis (Green et al., 2000). The fact that the plastids “stolen” by *Elysia chlorotica* still manage to function once ingested is particularly significant: how can plastids continue to perform photosynthesis in the absence of algal cytoplasm? There are three possible explanations: (1) either the *Vaucheria litorea* chloroplasts maintain their genetic and metabolic autonomy by producing autonomously all the metabolic resources to perform photosynthesis; (2) cryptic algal products (e.g., DNA, RNA and functional proteins) persist for a long time and can be used by plastids; (3) the mollusc partially contributes to this process by providing some of these metabolic resources. Several lines of evidence support the third hypothesis. First of all, the plastid genome does not contain all the protein-coding genes to satisfy its metabolic needs. Only considering photosynthetic capacities, it has been shown that the plastid encodes only one (i.e., RuBisCo) of the 12 essential photosynthetic enzymes (Rumpho et al., 2011, p. 307). Secondly, plastids are able to maintain functionality for several months. How they manage to do this is puzzling because, in order to maintain functionality, the plastids need to repair and substitute damaged proteins; when living inside *Vaucheria litorea* cells, the plastid can perform this process by exchanging DNA and functional proteins with the algal nucleus; however, the plastid cannot interact with the algal nucleus because it lives intra-cellularly in the animal’s digestive cells. Thirdly, there is evidence showing *de novo* synthesis of essential proteins in sea slugs starved for several months of their algal diet. Particularly this latter line of evidence suggests that the third hypothesis is correct. The evidence shows that the process is quite convoluted: it involves transfer of DNA sequences from algae to slug and, then, transfer of functional proteins from slug/host to plastid/symbiont. It is significant in this sense that the plastids seem to have relinquished two of the four layers of their membrane, which might render protein transfer more likely (Rumpho et al., 2011, p. 306). For instance, an enzyme synthesised from the nuclear oxygenated photosynthesis gene, *psbO*, is expressed in the sea slug and then likely exported to the plastid (Rumpho et al., 2011, p. 307). The DNA sequences from which the enzymes of the photosynthetic pathway are biosynthesised have been acquired on an evolutionary time scale by the animal *via* lateral DNA transfer, but the encoded proteins are produced, on an ontogenetic time scale, by the slug and exported to the plastid (Rumpho et al., 2008). Furthermore, the plastid photosynthetic pathways are reorganised. For instance, one of these – i.e., the Calvin-Benson cycle – involves a dozen of enzymes only three of which (RuBisCo and two other enzymes) are unique to phototrophs, having no known homologues in animals (Rumpho et al., 2011, p. 307, Figure 5). This means that the plastid performs the Calvin-Benson cycle by assimilating proteins either encoded by or imported from the slug, reorganising an old pathway by deploying enzymes assimilated through biotic entrenchment.

The developmental events characterising the establishment of the set of complex mutual ontogenetic dependencies between *Elysia chlorotica* and *Vaucheria*’s plastids on which we shall focus are three. First, proteins biosynthesised by the slug are assimilated by the plastid; to be realised, this process requires compatibility between the mechanisms of export and import on the parts of the partners, for instance, involving the simplification of the plastid membrane tailored to the acquisition of the proteins. Another form of cellular integration works in the opposite direction: the slug extracts the *Vaucheria*’s plastids and then functionally integrates them in the specialised tubular cells of its digestive epithelium. Thirdly, the proteins that the plastid assimilates from the slug are deployed in order to functionally re-organise the photosynthetic pathways with which the plastid contributes to the slug’s nutritional requirements. Note that the relevance of this example is not that the partnership between *Elysia chlorotica* and plastids is a consolidated mutualistic symbiosis like that, for instance, exhibited by eukaryotic cells or *Paulinella chromatophora* (see below). Its significance is rather that it is a partnership in the making, allowing us to unpack in some detail the ontogenetic dynamics of the process of entrenchment, that is, the kinds of functional integration (i.e., genomic, metabolic and cellular) occurring between partners and leading to the establishment of their mutual ontogenetic dependence.

These two different examples of biotic entrenchment identify various ways in which partners establish genomical, metabolic, and cellular irreversible ontogenetic dependencies. It is now time to consider how an autopoietic approach might make sense of this interactive and horizontal dimension of biological organisation.

## A Characterisation of Closure of Constraints in the Light of Entrenchment

The first step of our analysis is to dismiss, in sections “Can Set S Be Extended by Assimilating Components From the Environment?” and “Is Topology T Ontogenetically Invariant?”, some of the characterisations of closure that are in our view incompatible with entrenchment. This exercise is important in order to understand the ways in which closure of constraints is an interpretation of autopoiesis able to account for entrenchment.

In order to achieve our analytic aims, we propose a framework of analysis distinguishing composition, topology, and orchestration (see Vecchi et al., 2019). A set of entities S constitutes, as component parts, a biological system. The entities of the set S are spatially arranged according to a particular topology of interactions T. The entities of set S with topology T are causally organised in a particular relational fashion, functional organisation or orchestration O whereby the component parts perform particular activities that causally determine the behaviour of the biological system^10^. Now, we can pose and provide an answer to the following three questions related to ontogenetic biotic entrenchment:

1. Can set S be extended by assimilating components from the environment?
2. Is topology T ontogenetically invariant?
3. Can components assimilated from the environment become integrated in the functional organisation O and perform a function?

### Can Set S Be Extended by Assimilating Components From the Environment?

This first question concerns *components* closure. If closure is interpreted as internal strict self-production, the assimilation of components from the environment does not respect closure. It is uncontroversial that autopoiesis has been often interpreted as a claim about self-production, specifically in terms of circularity of production relations (Razeto-Barry, 2012). Obviously, there is a trivial sense in which this interpretation is meaningless, as it is invariably acknowledged that biological systems are thermodynamically open (see section “Biological Individuality Between Closure and Entrenchment”). Furthermore, it has been continuously recognised that self-maintenance occurs in spite of the continuous replacement of the *token* material components of which the biological system is constituted. *Components closure* is surely compatible with some form of transformation. Most obviously, given that *token* components are constantly destroyed, regenerated, environmentally assimilated etc., *components closure* is a claim about a self-produced subset of *types* of components^11^. Views concerning self-production vary extensively in the literature. At the extreme, Luisi (2003) defines autopoiesis as the thesis that all the molecular components for self-maintenance are internally produced, a view that is biologically unfeasible in the light of chemical and biotic entrenchment^12^. Some form of qualification is thus needed to characterise closure by identifying the subset of types of components that are, as a matter of fact, self-produced. Some authors (Cárdenas et al., 2010, p. 80) have proposed the idea of metabolic closure (labelled “enzymatic” by Mossio and Moreno, 2010, p. 278): “…all catalysts are synthesised internally; none is produced by any external agency^13^.” There are good empirical and theoretical reasons to challenge this characterisation of closure. Several lines of evidence indicate that the notion of enzymatic closure is problematic. Basically, enzyme production might be outsourced if appropriate mechanisms of protein-exchange evolve. Bacteria can transport proteins into the host cell’s cytosol *via* specific “needles” (e.g., type III secretion system). Usually the literature emphasises the transport of pathogenic proteins (Lu and Walker, 2001), but of course it could be used for beneficial (to the host cell) ones. Secondly, protein import/export between mitochondria or chloroplasts and host is well known (Poyton et al., 1992). In section “Mutual Dependence Through Genomic, Metabolic and Cellular Integration,” we saw that plastids import proteins from *Elysia chlorotica* and that have evolved a narrower membrane to facilitate import. A similar example of import of proteins concerns *Paulinella chromatophora*. This organism has two endosymbiotic chromatophores unable to reproduce alone, like mitochondrial and plastid organelles. On the one hand, on an evolutionary time scale, there is strong evidence in favour of the transfer of chromatophore DNA sequences to host. On the other hand, on an ontogenetic time scale, cytosol-synthesised proteins are imported back into the endosymbionts by releasing them into the inter-membrane space and crossing the peptidoglycan wall thanks to “…low molecular weights and nearly neutral charges, which probably represent adaptations to facilitate this passage.” (Bodył et al., 2012). Analogously to the cases of protein exchange between slug and plastid and between *Paulinella chromatophora* and chromatophore endosymbiont, most of the proteins required by mitochondria are encoded in the nucleus and imported into the organelle (Kuroiwa et al., 2006). All this evidence provides a proof of principle that the transfer of proteins between partners can evolve^14^. From a theoretical point of view, we agree with Razeto-Barry (2012) that self-production is conditional on what is available in the environment. What autopoietic systems do is, of course, to produce a subset of the types of components of the system, even though the subset actually produced depends contingently on what is available in the environment. Within this perspective, self-maintenance is achieved when the self-produced subset is:

“…capable of procuring the internal presence of the rest of the components of the system from the exterior, bringing them ‘in’ and maintaining them in a sufficiently local proximity to compose a physical unit. That is, it is not especially relevant whether the functional components of the system (we may exclude the ‘waste’) come from the environment or are produced internally, what is important is that they be present …” (Razeto-Barry, 2012, pp. 554–555)

More generally, entrenchment is inherently opportunistic and, arguably, the assimilation of components has no potential limits. Even though obviously there are no organisms that do not produce some of their token components, to postulate the invariance of some self-produced subset of types of components might turn out to be incompatible with entrenchment. The externalist ethos of entrenchment is, in any case, compatible with that proposed by the closure of constraints interpretation, whose focus is self-maintenance rather than self-production (Moreno and Mossio, 2015, note 7, p. 5)^15^.

### Is Topology T Ontogenetically Invariant?

Consider the case in which a biological system is constituted by a set of types of component entities S, some of which are self-produced and some of which are assimilated from the environment, contravening the interpretation of autopoiesis as strict self-production. We can now ask whether this system of components must respect a specific spatial arrangement or topology in order to be considered that very system and ground individuality ascriptions.

At first sight, characterising closure in terms of topological invariance is counterintuitive given that biological systems continuously undergo a series of ontogenetic structural changes. However, in a more interesting and restricted sense, ontogenetic change is compatible with some form of topological invariance; when the claim is made that some metabolic pathways such as those at the core of protein biosynthesis are invariant and unaltered despite assimilation of environmental components (e.g., lateral DNA transfer, amino acid transfer and cofactors exchange between host and endosymbiota, Wernegreen, 2012), a more circumscribed claim concerning invariance is made. Topological invariance is in fact compatible with the externalism of entrenchment characterised in section “Can Set S Be Extended by Assimilating Components From the Environment?”, in the sense that the rationale of the characterisation of closure in metabolic and enzymatic terms might be to capture a topological invariant of biological organisation rather than self-production. Nonetheless, it seems to us that the assimilation of metabolic interactants or enzymes from one of the partners allows some form of plasticity, for instance in the form of the transformation of the topology of an old metabolic pathway. Consider a case in which an organism produces a component part in a 4-step series of biochemical reactions whereby the end product of the previous reaction is the starting product of the following; the topology of the network of production consists of a cycle with four reactions; suppose the organism starts assimilating a reactant from the environment which allows the simplification of the network by bypassing one of the steps, for instance by reducing the production chain to three biochemical reactions; in this case, which is structurally similar to that illustrated in section “Dependence Through Division of Metabolic Labour,” a transformation of the topology of the network of production or metabolic pathway ensues. An example of transformation has been illustrated in section “Mutual Dependence Through Genomic, Metabolic, and Cellular Integration”: the Calvin-Benson cycle involves a dozen enzymes, most of which are proteins either encoded by or imported from the slug; when slug-biosynthesised enzymes are assimilated by plastids and deployed in order to re-organise their photosynthetic pathway, possible variations in the topology of the pathway might ensue.

More generally, we concur with Razeto-Barry (2012, p. 557) that biological systems such as organisms do not primarily aim to resist changes to their organisation and preserve some aspect of topology invariant throughout ontogeny; they rather strive to preserve their spatio-temporal unity or numerical identity in spite of the variety of physico-chemical changes they are subjected to. Additionally, we would like to add that biological systems are opportunistic so that any assimilated environmental component might be, when available and whenever possible, functionally entrenched in order to preserve their identity. Perhaps some form of topological invariance might characterise biological systems at some level of analysis but, given the above considerations, the search for significant topological invariants encompassing all kinds of organisms is probably futile. Unless the closure of constraints interpretation is committed to this search, we do not see any reason why it should be unable to account for the ontogenetic changes generated by entrenchment. After all, the claim is frequently made in this literature that structural (as well as functional) variation is not simply an obstacle to maintaining biological organisation, but a crucial condition for adaptability (Ruiz-Mirazo and Moreno, 2004; Montévil and Mossio, 2015, p. 190).

### Can Components Assimilated From the Environment Become Integrated in the Functional Organisation O and Perform a Function?

The upshot of the two previous sections is that biological systems are both compositionally and topologically “open” during ontogeny. The question we need to ask now is whether the components assimilated from the environment can become integrated into the functional organisation or orchestration of the biological system and be ascribed a function. This is a crucial issue because, for instance, Queller and Strassmann (2016, p. 861) suggest that “Acquiring a symbiont that has already perfected certain functions on its own can be by far the most rapid way of acquiring novel functionality”. The issue of this subsection is how a closure of constraints interpretation can make sense of claims of this kind.

In the context of the organisational approach, the concept of function pertains to current (rather than evolutionary) biological organisation; given that biological organisation is characterised in terms of closure of constraints, it follows that the concept of closure grounds functionality within biological systems: “…constraints subject to closure constitute biological functions” (Montévil and Mossio, 2015, p. 186). Applied to entrenchment, the idea is that components assimilated from the environment, when maintained within that organisation, can acquire and thus, be ascribed, a function only if they make a specific contribution as a constraint to the self-maintenance of the system itself. For instance, a component could act as a catalyst in a metabolic reaction or modify the topology of a metabolic pathway. In all these cases, the acquisition of a function within the organisation of the system on the part of an assimilated component is the end result of ontogenetic entrenchment^16^. In order to see whether entrenchment and closure of constraints are compatible, let us take a look again at our examples.

The example illustrated in section “Dependence Through Division of Metabolic Labour” is unproblematic for an account of closure of constraints. As we stressed, following developmental events like genome reduction and loss of catalase-peroxidase, the dependence between Ps and Hs is *ontogenetically irreversible* in the sense that, in order to survive, Ps must assimilate and functionally integrate deoxygenated water. Such developmental events cause the structural and functional variations undergone by Ps and, specifically, the loss of the use of the metabolic pathway where catalase-peroxidase “controls” water deoxygenation. As we suggested, these ontogenetic dynamics can be characterised in terms of a change in the subset of constraints realising closure, more specifically in terms of a loss of constraint, which implies an organisational transformation of the sets of constraints realising closure. As we anticipated in section “Biological Individuality Between Closure and Entrenchment,” one of the problems posed by the dynamics of biotic entrenchment characterising multi-species partnerships is that it becomes difficult to discriminate for which of the entrenched biological systems closure is realised. It could be argued that in the case at hand, whereby an ontogenetically irreversible dependence between Ps and Hs is at stake, what realises closure is actually their partnership. On the contrary, we would argue that in this specific case the partnership is not the entity realising closure and cannot be treated, as a consequence, as an encompassing or extended biological individual. The reasons are the following. It has been argued that the autopoietic approach provides the basis for an extended definition of life and cognition and, concomitantly, for a reinterpretation of the notion of closure. For instance, Virgo et al. (2011) argue that worm’s digestion is an extra-organismic process amenable to be understood in extended autopoietic terms: the worm secretes in the external environment enzymes that decompose food and re-assimilates the products of this external decomposition, finally making use of them for its own metabolic purposes. The basic idea of the extended autopoietic approach is that closure is a property of the “extended organism” or, put differently, that, given that physiological and developmental processes are not *organism-bound*, they are neither *organism-centric*. This perspective has counter-intuitive implications, recently highlighted by Vecchi (2019). The problem of the extended approach in this specific case is that, apart from the fact that the above relationship is not mutual (P needs H but H does not need P), entrenchment remains, despite not being organism-bound, organism-centric^17^. In fact, P remains the location of the assimilation and functional integration of the externally produced deoxygenated water, processes that are organism-centric. Thus, there is a crucial distinction between the developmental system or organism P on the one hand and the partnership as an extended biological system or web of causal interactions between Ps and Hs on the other. Therefore, we argue that there is no problem in this case to think about closure as a property of Ps and Hs separately and, concomitantly, no reason to think that the partnership is an extended biological individual also realising closure^18^.

The same kind of analysis cannot, however, be applied to the example considered in section “Mutual Dependence Through Genomic, Metabolic, and Cellular Integration,” suggesting that an extended interpretation of autopoiesis is more legitimate. In cases of this kind, the process of functional integration is not clearly organism-centric but, rather, arguably *partnership-centric*. When partners exchange, recruit and re-deploy - on an ontogenetic time scale – genomic, metabolic and cellular resources, a complex range of mutual dependencies is established between them. As we have seen in section “Mutual Dependence Through Genomic, Metabolic, and Cellular Integration,” partners exchange both genomic resources through lateral DNA transfer (e.g., from plastid to *Elysia* through *Vaucheria*) and a variety of metabolic resources such as amino acids, vitamins, co-factors, co-enzymes (from plastid to *Elysia* and vice versa) and proteins (from *Elysia* to plastid, a process involving the simplification of the plastid membrane); they also recruit in metabolic pathways enzymes biosynthesised by the partner (as plastids do within *Elysia* by deploying such enzymes in order to functionally re-organise their photosynthetic pathways), have the means to functionally integrate entire cells within their bodies (as *Elysia* does by functionally integrating plastids in its specialised tubular cells of the digestive epithelium) and harness the metabolic capacities of endosymbionts (as *Elysia chlorotica* does by benefiting from plastids’ photosynthetic capacities in order to satisfy its nutritional requirements). The existence of this complex range of mutual dependencies of increasing genomic, metabolic and cellular integration makes it difficult to deny that the extended approach might have a valid point at this juncture. The chief reason is that partners exhibit these latter causal capacities only within the partnership, in the sense that such causal capacities would not be instantiated in other relational contexts. For instance, it is only within the context of the orchestration of the encompassing system constituting the partnership that *Elysia chlorotica* can functionally integrate plastids in its specialised tubular cells of the digestive epithelium. Conversely, it is only within the context of the orchestration of the encompassing system constituting the partnership that plastids, for instance, relinquish two layers of their membranes in order to facilitate proteins import from the slug^19^. Above all, *Elysia chlorotica* can become a photosynthetic animal only by interacting appropriately with plastids within a specific relational context or biological orchestration. Given that it is only within the encompassing system or partnership that partners behave in particular ways accounting for the mutual dependence dynamics of the partners, the encompassing system possesses what could be called “global constraints” controlling, harnessing and channelling the behaviour of the partners. We might thus say that the complex web of give and take interactions, irreversible ontogenetic mutual dependencies and functional exchanges characterising cases of biotic entrenchment of this kind is better seen as *a merging of constraints systems* whereby the functional and physical boundaries of the partners become progressively more difficult to discriminate. Moreno and Mossio (2015, p. 23) argue that: “… by relying on closure, the autonomous perspective clearly favors … functional criteria over physical ones to define the boundaries of biological organisms.” Approaches like Villalobos and Razeto-Barry (2019) based on the distinction between embodied and embedded living beings (see footnote 18) seem to equate functional and physical boundaries and ground the former in terms of the latter: the embodied living system is physically separated and functional integration can be fully accounted from its perspective. The photosynthetic slug case is instructive in this sense because it is difficult to equate the functional boundaries of the partnership with the physical boundaries of the partners at all stages of ontogenesis given that the plastids are not part of the entire life history of the slug and vice versa. Most importantly, the photosynthetic slug case is instructive because this partnership-in-the-making provides some clues on how complex mutual dependencies might evolve into mutualistic symbioses^20^. The more the ontogenetic dependencies caused by biotic entrenchment occur within a physical boundary, the more the embodied and organism-centric perspective can account for them. At the extreme, such as the case of *Paulinella chromatophora* (section “Can Set S Be Extended by Assimilating Components From the Environment?”) and chromatophores or, indeed, eukaryotic cells and organelles, partnerships constitute physically-separated and embodied wholes for the entire ontogenetic process. But this is an evolutionary achievement, the pinnacle of the entrenchment process generating, as entrenched outcomes, new kinds of organisms. Compatibly with our analysis, in the evolutionary literature such phenomena are understood in terms of gradually increasing levels of organismality that, at the extreme, can be seen as transitions in individuality (Queller and Strassmann, 2016).

Thus, the dynamics of functional integration at the heart of biotic entrenchment in the case of partnerships generates a foundational problem for any account of closure because it is difficult to discriminate the system of constraints of two partners with precision when irreversible ontogenetic dependencies and functional exchanges are so intertwined. In cases of merging of systems of constraints, such dynamics are not merely accountable in terms of loss of constraints as in the case illustrated in section “Dependence Through Division of Metabolic Labour.” They also require a further significant modification of the closure of constraints account of biological organisation. When the organisms in a multi-species partnership develop irreversible ontogenetic mutual dependencies such that they rely on the partners for their own physiological maintenance, that is, when their self-maintenance is not reducible to the internal dynamics of the system of constitutive constraints (those realised within the spatial boundary of the biological system’s organisation), realising closure depends on the existence of external constraints (those realised between the biological system and the environment through biotic entrenchment, more specifically between biological systems through merging of their constraints systems). Thus, does closure belong to partners, the partnership or both? Montévil and Mossio (2015) approach this problem in these terms. Suppose two cells establish irreversible ontogenetic direct and mutual dependencies; as a consequence, the encompassing system realises closure: “In this situation, is there a legitimate way to argue that the individual interacting cells also realise closure?” (Montévil and Mossio, 2015, p. 188). This thought experiment straightforwardly applies to the photosynthetic slug case. In order to answer the above question, these authors propose a formal solution by means of which the two cells can be “represented as two discriminable systems”. Does this mean that they realise closure? Montevil and Mossio clarify that, even though the formal procedure does not allow to discriminate between the systems in terms of *strict discontinuities*, they can be drawn in terms of a *tendency to closure*, that is, a measure of the organised complexity of the cells that can be used to represent the degree of organisational integration between them:

“The tendency to closure is a measure of the degree of organisational integration of organisms and, as well as, an operational tool for drawing the boundaries between them, even when they establish functional dependence. It is worth emphasizing, in this respect, that such a measure comes in degrees.” (Montévil and Mossio, 2015, p. 189)

Tendency to closure is a notion capturing some of the peculiarities of biotic entrenchment, i.e., the difficulty to separate in terms of *strict discontinuities* the functional boundaries between partners whereby systems of constitutive and external constraints are merging. One of our chief aims in this paper is to translate the notion of tendency to closure proposed by Montévil and Mossio to biologically realistic cases. Montévil and Mossio (2015, see Figure 1, p. 189) only consider a simplified case where the degree of organised complexity of the cells is higher than that of the encompassing system. But such cases are, in our opinion, certainly not the more interesting and challenging for closure of constraints accounts. The photosynthetic slug case is especially interesting because it shows that the organised complexity of the partnership is higher than that of the partners taken individually; the reason, again, is that the partnership possesses a rich set of global constraints controlling, harnessing and channelling the behaviour of the partners. A biologically feasible, rather than merely formal, interpretation of the notion of tendency to closure makes sense of the existence, in the biological world, of multi-species partnerships with varying degrees of functional dependence between partners and gradually richer sets of global constraints, making closure ascriptions to the partnership progressively more realistic from a biological point of view. The concept of tendency to closure also makes sense of the claim that partnerships are outcomes of the process of biotic entrenchment, with the emergence of new biological individuals at the extreme^21^.

The case of partnership thus shows that components assimilated from the environment can become functionally integrated or entrenched in a variety of ways by the partners. At one end of the continuous spectrum of the dynamics of functional integration establishing ontogenetic dependencies between partners, we have cases where it is unproblematic to take an organism-centric perspective and dismiss the suggestion that it is the partnership that realises closure (i.e., being the extended biological individual). But, at the other end, the suggestion stemming from the extended approach to autopoiesis (i.e., that it is the partnership the system that realises closure) gains strength because the dynamics of functional integration establishing ontogenetic dependencies between partners cannot be fully understood from the organism-centric perspective. In the latter cases, a merging of constraints systems might characterise such dynamics in terms of the loss but also assimilation of further external and non-constitutive constraints, in terms of the progressive internalisation of such external constraints on the part of the partnership and in terms of the generation of global constraints. Finally, at the extreme of the spectrum, the diachronic emergence of new kinds of organisms occurs as the result of the interactive construction of biological individuality through the progressive transformation of the horizontal organisation achieved through entrenchment-driven merging of constraints into vertical complexity (i.e., the hierarchical organisation of parts making up a whole). The more the systems of constraints are merged, the more closure and individuality ascriptions to the partnership make sense. Thus, biotic entrenchment implies that the biological individuality of the biological system might be constructed *via* the interaction with the other biological systems present in the environment. It is in this sense a chief example of interactive construction of biological individuality and inter-identity.

## A Characterisation of Organisational Invariance in the Light of Entrenchment

Another aspect of the problem posed by entrenchment for autopoietic accounts based on closure is the characterisation of the concept of organisational invariance. If closure of constraints does not capture an invariant aspect of biological organisation, then it has no genuine explanatory role in biology (Moreno and Mossio, 2015, p. 2). We have seen that entrenchment potentially implies, on an ontogenetic time scale, compositional openness (section “Can Set S Be Extended by Assimilating Components From the Environment?”) and plasticity of biological organisation (“Is Topology T Ontogenetically Invariant?” and “Can Components Assimilated From the Environment Become Integrated in the Functional Organisation O and Perform a Function?”). Self-maintenance potentially occurs despite the continuous material, structural and functional ontogenetic changes partially caused by biotic entrenchment. How do we account for the preservation of the identity of the biological system under consideration? What is the measure of organisational invariance that can be used in order to individuate a system as that very system? The implicit answer to this question is provided by the analysis of the previous sections. We suggest that closure is a *meta-property of biological individuals*: rather than stressing the invariance of a specific aspect of biological organisation (i.e., a specific system of constraints), this interpretation acknowledges the plasticity of the system of constraints. Thus, we argue that what remains fixed during ontogeny is merely the circularity of the network’s operations rather than the physical nature of the components, or rather than the topology of the network of components’ interactions or even rather than the functional orchestration of the biological system. Closure thus specifically refers to the invariance and ontogenetic stability or conservation of the circular organisational pattern between the component parts of the orchestration. This pattern constancy grounds the numerical identity of the biological system.

Is our interpretation of closure in the light of entrenchment compatible with other closure of constraints interpretations? As far as we can see, we can identify two central claims of the closure of constraints interpretation. The first is that, given that the concept of constraint is relativised to particular temporal and spatial scales as well as to a specific processual context, “…closure is a multiscale causal regime…” (Montévil and Mossio, 2015, p. 187). The second is that any individual biological system, despite undergoing a variety of ontogenetic changes, maintains some form of closure of constraints that is realised in different variants by adding or suppressing specific constraints or sets of constraints. We interpret this point as meaning that the invariance of closure instantiated by an individual system is not characterisable in terms of a fixed and specific pattern of mutual dependence between a subset of specific and fixed constitutive constraints. As the example in section “Dependence Through Division of Metabolic Labour” shows, suppression of one constraint is not enough for loss of closure and identity. Equally, as the example in “Mutual Dependence Through Genomic, Metabolic, and Cellular Integration” shows, addition of constraints does not necessarily mean loss of closure of the individual partners, but rather poses a question concerning the possibility that the partnership or encompassing system realises some form of closure. It is for this reason that we suggest to interpret closure of constraints compatibly with our interpretation, as referring to a type of circular organisational pattern that remains constant, precisely the pattern of mutual dependence between a changeable subset of constraints. Indeed, Montévil and Mossio (2015, p. 190) seem to argue along similar lines: “…the invariance of closure takes place at a level of description which is higher than that at which each specific organisation (instantiated by an individual system) occurs” (Mossio et al., 2016) Thus, closure could be either seen as a *meta-property* realised by all biological individuals or as the *circular organisational pattern between the component parts of the orchestration* grounding the numerical identity of biological individuals, that is, as an invariant of biological organisation that, notwithstanding entrenchment, allows a possible solution to the problem of biological identity (as seemingly argued by DiFrisco and Mossio, 2019). Unlike an autopoiesis in the traditional sense of the term (i.e., strict self-production), autopoiesis in the light of entrenchment does not require the internal production of all the necessary components to maintain the circularity of processes and patterns that support the individual’s numerical identity, nor does it require the production of a set of specific elements or functions for the biological organisation to persist. What the individual requires is to maintain its circular organisation by exploiting any suitable environmental resource, which in a stable ecological context eventually might imply the internalisation of environmental elements. Whether such an interpretation of closure is sufficiently strong to ground biological individuality ascriptions is a chief challenge for autopoietic approaches.

## Author Contributions

All authors listed have made a substantial, direct and intellectual contribution to the work, and approved it for publication.

### Conflict of Interest

The authors declare that the research was conducted in the absence of any commercial or financial relationships that could be construed as a potential conflict of interest.

## Footnotes

^1^Physiological and evolutionary accounts are complementary and, sometimes, integrative. For instance, Queller and Strassmann (2016) characterise individuality as the achievement of *functional adaptive coherence* or “organismality,” a property of biological systems that is not categorical but continuous. Conversely, some physiological accounts take into account the evolutionary dimension of biological individuality, especially insofar as the origin of new organisations is concerned (Moreno and Mossio, 2015).

^2^Even though there exists some affinity between West-Eberhard’s concept and the concepts of generative entrenchment (Wimsatt, 1986) and developmental scaffold (Wimsatt and Griesemer, 2007), we shall focus solely on the first.

^3^Interestingly, in one of the rare references to autopoiesis in this literature, Gilbert et al. (2010, p. 673) suggest that, given “interspecies epigenesis,” the fertilised egg is a “symbiopoietic” rather than an autopoietic, self-creating, entity.

^4^Part of the biological literature focuses on the evolution of functional adaptive coherence and “organismality” (Pepper and Herron, 2008; Queller and Strassmann, 2016). Functional adaptive coherence can be characterised as the degree to which partners work together to function as a unit without conflict, as the capacity of becoming consolidated units of function on an evolutionarily time scale. Thus, partners’ dependency comes in degrees. In contrast, as we shall see, closure is a categorical property that is either realised or not. In this sense, a putative contrast between evolutionary and physiological accounts comes to the fore.

^5^For instance, a molecule of “…ATP is not a constraint for a reaction that uses its energy (it is consumed): however, it is a constraint for the transformation and transport of the energy of glycolysis (or another reaction) to a target reaction, since this process leaves ATP invariant.” (Montévil and Mossio, 2015, p. 184, note 18).

^6^We characterise a developmental event as a cause of a qualitative change to an individual organism. This characterisation draws inspiration from the analysis provided by Mahner and Bunge (1997, pp. 313–316). These authors reduce evolution to a series of developmental events of “ontological speciation”, a thesis that we reject because inheritance (for instance through reproduction) should be taken into account in order to understand evolutionary dynamics.

^7^Biotic entrenchment in the evolutionary sense is at the root of, for instance, the origin of life and eukaryogenesis: in both cases, an original biological system assimilates environmental elements available in the environment and gradually functionally integrates them within its organisation, eventually generating, on an evolutionary time-scale, a different biological individual.

^8^A comprehensive evolutionary account encompassing both unicellular and multicellular cases is that proposed by Queller and Strassmann (2016). This account – that we find particularly insightful – is tailored to understand how “… multi-species aggregates can be considered to be consolidated units of function, with very high cooperation and very low conflict amongst their parts.” (Queller and Strassmann, 2016, p. 859). Significantly, Queller and Strassmann argue that, even though multispecies partnerships are common in nature, the ontogenetic relationship between partners might be evolutionary transient, with the consequence that transitions in individuality might be scarce. Importantly, Queller and Strassmann think of the stability and durability of the partnership in compositional rather than merely functional terms and are sceptical that functional stability is enough for higher levels of organismality (Queller and Strassmann, 2016, p. 868). Given that biofilms, holobionts and, more generally, ecosystems are most often characterised by functional stability but compositional fluidity, they do not generally possess a high level of organismality.

^9^However, the question of when a developing organism becomes, during ontogeny, an autonomous individual might not be answered by invoking the general definition of development we endorse. We thank a reviewer for highlighting this point.

^10^The distinction between topology and functional organisation is analytic given that topology is an abstraction. In biochemistry, similar distinctions are used in order to discriminate the topology and architecture of a protein from its orchestration (i.e., the folded and functional protein). Component parts are both individual parts and structures composed of individual parts but smaller than O. So, if individual parts are amino acids and if O is a cell, a protein is a component part because it is a structure composed of amino acids that is smaller than the cell.

^11^If, on the other hand, a subset of token components is invariant ontogenetically (not subject to transformation), *components closure* would potentially provide an essentialist way of characterising the biological individual.

^12^Additionally, as one reviewer highlighted, many intracellular components are inherited. For instance, when cells divide, some functional ribosomes pass to the daughter cell. This example of material and non-genetic inheritance is an instance of trans-generational biotic entrenchment.

^13^Intriguingly, Cornish-Bowden et al. (2007) argue that the distinction between metabolites and enzymes is spurious because many metabolites are enzymes and because catalysis is not a fundamental property of life, ironically undermining the position defended in the article here mentioned (Cárdenas et al., 2010). There are two reasons for this. The first (Cornish-Bowden et al., 2007, p. 2402) is that catalytic enzymes are not necessary for chemical reactions: in the urea cycle, what is necessary is that the cycle of chemical reactions is “invariant”; no catalytic enzymes are necessary to regulate this cycle, even though without them the chemical reactions are much slower; thus, the urea cycle can be given an interpretation purely in terms of chemical transformations by avoiding reference to catalysis, capturing the essence of the process. The second reason (Cornish-Bowden et al., 2007, p. 2403) is that, from an evolutionary perspective, enzymatic catalysis is an acquired phenotype and that chemical reactions were likely performed by much simpler molecules than proteins.

^14^Of course, plastids, chromatophores and mitochondria are not “organisms,” even though, by assumption, they were. But the point here is that it would not be surprising to find cases of protein exchange in the opposite direction, from organelles to unicellular or multicellular organism.

^15^Some form of bias still permeates the closure of constraints literature. For instance, discussing the origin of functional differentiation characterising primitive biological systems, Moreno and Ruiz-Mirazo (2009, p. 596) argue: “…what is important to stress is the fact that these diverse constraints should mutually enable their continuous regeneration, in a way that it is possible to start saying that the self-maintaining system is endogenously producing—and reproducing—all of them. This is crucial not only for robustness, but also for the very emergence of a functional domain: by internally synthesising its own constraints the system becomes much more plastic; i.e., capable to perform a diversified modulation of its own self-maintaining dynamics.” Moreno and Ruiz-Mirazo seem to assume that the process of generation of a new functional component can only be strict self-production, whilst the obvious alternative is that it was assimilated from the environment.

^16^Again, we stress that this process of functional integration is ontogenetic in the sense that it must be caused by developmental events occurring to individual organisms. With this we do not deny that functional integration has an evolutionary dimension involving inheritance events (which are at the basis of the increasing gradual genomic, metabolic and cellular integration of partners), but just that it must also be developmental events that account for the fact that at some point in time a component assimilated from the environment becomes functionally integrated within the organisation of the partner.

^17^The only reason such univocal relationship persists is that Hs are ubiquitous in Ps natural environment. If Hs were not omnipresent, Ps would eventually re-acquire, through mutation or lateral gene transfer, the *katG* genes. Otherwise they would become extinct.

^18^Analogously, Villalobos and Razeto-Barry (2019) also reject the extended autopoietic interpretation by distinguishing *embodied* and *embedded* living beings. Translated to our case, Ps are the embodied organisms functionally integrating the ontogenetic resources produced by Hs; whilst their partnership is an embedded system that is clearly not organism-bound but that at the same time cannot be treated as an extended organism or individual; closure ascription to the embedded system is unwarranted in cases akin to that illustrated in “Dependence Through Division of Metabolic Labour.”

^19^The fact that plastids are not sufficiently organismal (hence not amenable to closure ascriptions) is irrelevant for the general point made at this juncture. What is important is that the encompassing system or partnership is amenable to closure ascription despite the lack of closure of one of the partners.

^20^One of the reviewers suggests that the relationship between *Elysia chlorotica* and plastids is analogous to the relation between a multicellular organism and its cells, which can be accounted for in terms of “nested closures” (Montévil and Mossio, 2015). According to this view, cells taken individually do not contribute as constraints to the maintenance of the multicellular system because, first, the individual cell’s effect on maintenance processes is negligible and, secondly, cells act as constraints only collectively. The upshot of this analysis is that there is a clear separation between the closure of the individual cells and the closure of the multicellular organism in such cases. But this interpretation of the relationship between *Elysia chlorotica* and the plurality of plastids assimilated by the slug is in our opinion problematic. First of all, it must be the case that individual plastids act as constraints by, for instance, transferring genomic or metabolic resources to *Elysia chlorotica,* exchanges without which the slug would not become a photosynthetic animal. Secondly, plastids do not seem to behave collectively but individually as constraints. It is because of the various exchanges of genomic and metabolic resources between *Elysia chlorotica* and the plurality of plastids that the partnership they constitute can be considered as an encompassing system for which closure ascriptions might be legitimate.

^21^A related foundational issue is whether, in the case of multi-species partnerships, closure is always maintained by either the partnership or partners despite the functional exchanges between the latter. This is the view defended by closure of constraints advocates: “…the claim according to which closure constitutes an ‘invariant’ of biological organisation technically means that a description of closure is possible for any interval long enough to describe a sufficient set of constraints and their mutual dependencies. In other words, given a minimum duration, closure is realised for any interval of equivalent duration chosen in the system’s lifetime.” (Montévil and Mossio, 2015, p. 190; see also Moreno and Mossio, 2015, p. 24). We reserve treatment of this complex issue for another article.
